# AI-Driven Plant-Derived Anti-Infectives: Integrating Traditional Wisdom into Precision Medicine Against AMR

**DOI:** 10.3390/life16040540

**Published:** 2026-03-25

**Authors:** Zhiwu Yin, Changbin Chen, Xing Wu, Wenhao Luo, Paulo Quaresma, Jianbiao Dai

**Affiliations:** 1 Netclass Technology Inc., Shanghai 200072, China; yinzhiwu@netclasstech.com; 2 Academy for Smart Health and Longevity R&D, University of Saint Joseph, Macao SAR, China; luo.wenhao@usj.edu.mo; 3 Shanghai Institute of Immunity and Infection, Chinese Academy of Sciences, Shanghai 201203, China; cbchen@ips.ac.cn; 4 School of Computer Engineering and Science, Shanghai University, Shanghai 200444, China; xingwu@shu.edu.cn; 5 Computer Science Department, School of Sciences and Technology, University of Évora, Rua Romão Ramalho 59, 7000-671 Évora, Portugal; pq@uevora.pt

**Keywords:** artificial intelligence, natural products, antimicrobial resistance, mechanism of action elucidation, ethnobotany, anti-infective drug discovery, computational pharmacology

## Abstract

The escalating antimicrobial resistance (AMR) crisis necessitates the development of innovative anti-infectives with novel mechanisms of action. Nevertheless, research on natural products remains constrained by low-throughput screening and limited mechanistic insights. Artificial intelligence (AI) is catalyzing a pivotal paradigm shift—from the mere isolation of active compounds to precisely deciphering their modes of action. This review highlights AI’s transformative role in bridging ethnopharmacological knowledge and modern pharmacology to decode the mechanisms of plant-derived anti-infectives. Case studies on berberine, baicalein, danshensu derivatives, and rosmarinic acid derivatives from *Coleus amboinicus* illustrate AI’s capacity to map traditional therapeutic concepts to specific pathways (e.g., biofilm inhibition, inflammasome modulation) and to predict precise binding interactions and pharmacophores with high precision. Leveraging statistical correlations between ethnobotanical usage patterns and chemical similarity, we propose a “Knowledge–Data–Mechanism” three-layer framework centered on deep mechanistic insight. Integrating Chinese initiatives, such as the CNDR (China’s National Drug Repository) database and the TCM-AI platform, with global traditional medicine wisdom, this strategy provides an actionable roadmap for modernizing anti-infective discovery. Validated applications of this paradigm have demonstrated order-of-magnitude acceleration in mechanistic characterization, rapidly yielding structurally novel agents with well-defined, target-specific actions—a critical advancement in addressing the urgent global threat of antimicrobial resistance.

## 1. Introduction

### 1.1. The AMR Crisis and the Dilemma in Anti-Infective Drug Development

Antimicrobial resistance (AMR), designated by the WHO as a critical global health threat of the 21st century, undermines medical safety across both routine procedures and critical care [1]. The proliferation of superbugs (e.g., MRSA [methicillin-resistant Staphylococcus aureus], CRE [carbapenem-resistant Enterobacteriaceae]) erodes antibiotic efficacy, thereby increasing the risks of untreatable infections [2]. Anti-infective development remains mired in a decades-long discovery void: only a few antibiotics with novel mechanisms have been approved (e.g., the oxazolidinone linezolid in 2000, the lipopeptide daptomycin in 2003, and, more recently, the siderophore cephalosporin cefiderocol in 2019), highlighting the stark contrast between pipeline depletion and accelerating resistance [3]. The principal bottleneck lies not merely in lead screening but, more critically, in delays in validating novel mechanisms of action [4]. Conventional high-throughput screening isolates active compounds but often fails to identify precise targets and pathways, prolonging mechanistic validation beyond bacterial evolutionary rates [5]. Consequently, innovative paradigms enabling efficient mechanism elucidation are urgently required to address the AMR crisis [6].

### 1.2. The Gift of Plants and the “Mechanistic Black Box” Bottleneck

Natural products remain indispensable in anti-infective discovery, with artemisinin and platensimycin exemplifying the therapeutic potential of plant-derived compounds [7]. Global traditional systems—Traditional Chinese Medicine (TCM) (“Coptis for dysentery, *Scutellaria* for pulmonary ailments”) [8], Ayurvedic formulations [9], and West African Agunmu remedies against malaria [10]—offer centuries of clinically validated wisdom. Ethnobotanical research confirms that phylogenetically related plants share phytochemical spaces, with structural similarity statistically correlating with therapeutic use [11], thereby forming the biological basis for AI-driven inference of bioactive molecules [12]. For example, Agunmu analysis identified kaempferol glycosides as anti-malarial agents via phenolic target interactions, validated in vitro and in vivo [10]; Samoan “*matalafi*” was decoded through omics approaches to reveal anti-inflammatory mechanisms [13].

Yet traditional knowledge remains an underexplored reservoir due to insufficient tools for mechanistic elucidation [14], causing promising leads to stall because of unknown mechanisms [15]. The classical “collection–extraction–fractionation” paradigm functions as a low-throughput “fishing expedition” [16], frequently neglecting pivotal druggability questions: mechanism, precise target, and resistance evasion [17]. This “mechanistic black box” impedes candidate translation and hinders assimilation of traditional pharmacological wisdom, exacerbating the global anti-infective development crisis outlined previously [18].

### 1.3. Paradigm Shift Driven by AI: From “Identifying Active Molecules” to “Elucidating Mechanisms of Action”

Artificial intelligence (AI) is catalyzing a paradigm shift in anti-infective discovery—from blind “fishing expeditions” to precise “intelligent navigation” [19], enabling computational formulation and validation of mechanistic hypotheses prior to wet-lab experimentation [20]. Advanced architectures, including graph neural networks (GNNs) and large language models (LLMs), integrate molecular structures, multi-omics data, and unstructured textual knowledge into a unified semantic space [21,22], shifting the focus from simple activity prediction to generating testable hypotheses on biological intervention mechanisms [23]. Global exemplars include halicin’s AI-predicted disruption of the bacterial proton motive force targeting CRE (carbapenem-resistant Enterobacteriaceae) [24]; knowledge graph–accelerated repurposing of baricitinib for COVID-19 [25]; and Phase IIa validation of AI-designed ISM001-055 (rentosertib) [26].

Conversely, the Phase I discontinuation of DSP-118 underscores the persistent gap between computational predictions and physiological complexity, reaffirming that “computational hypotheses” require rigorous experimental closed-loop validation [19,27]. Collectively, these cases establish a “Mechanism-First” paradigm [28], transforming research from passive compound identification to active elucidation of host–pathogen interactions and biological processes [29].

### 1.4. Framework and Core Contributions of This Review

**Figure 1:**
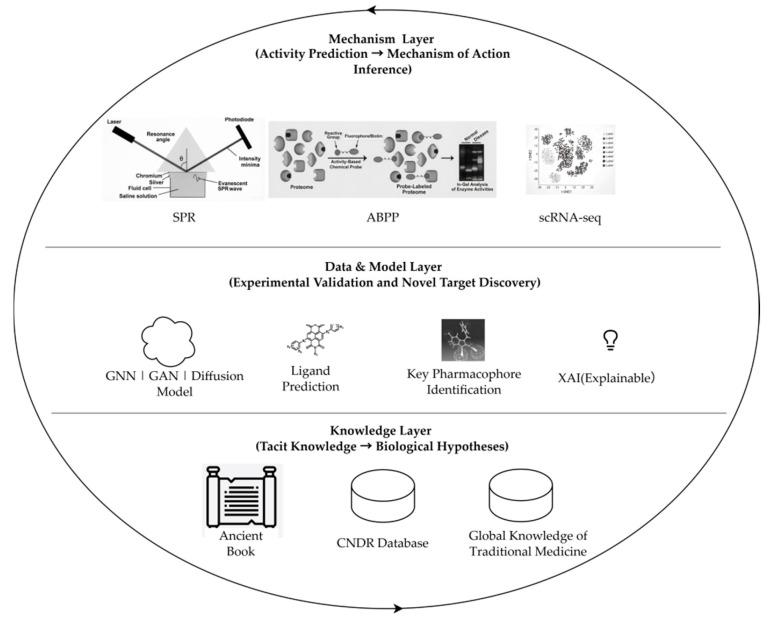
The “Knowledge–Data–Mechanism” three-layer integration framework centered on “mechanism elucidation”.

The primary objective of this review is to propose and critically examine a tripartite “Knowledge–Data–Mechanism” framework (Figure 1) designed to address a central question: How can we systematically decode the mechanistic basis of traditional medicine-derived anti-infective agents using artificial intelligence? This framework is centered on deep mechanistic dissection to unlock the anti-infective potential of plant-derived small molecules. As a forward-looking review, our principal contribution lies in synthesizing disparate advances—from knowledge graph construction to explainable AI and multi-omics validation—into a coherent integration logic specifically tailored for traditional medicine-guided anti-infective discovery. Unlike prior reviews that focus on individual technologies or compound classes [16,30], this framework demonstrates how these elements can function synergistically to transform empirical traditional knowledge into testable mechanistic hypotheses.

The Knowledge Layer leverages ethnopharmacological databases (e.g., CNDR) and natural language processing (NLP), guided by ethnobotanical correlation principles, to translate traditional concepts (e.g., TCM’s “clearing heat and detoxifying”) into precise biological pathways (e.g., NLRP3 inflammasome activation, biofilm inhibition), converting empirical wisdom into computable mechanistic hypotheses [31,32].

The Data and Model Layer integrates advanced algorithms—GNNs, GANs, and diffusion models—with explainable AI to move beyond activity prediction, identifying targets (e.g., PBP2a, MuDHFR), predicting binding modes and pharmacophores, and generating testable mechanistic hypotheses extendable to antiviral and antiparasitic contexts [33,34,35,36,37,38]. The Mechanism Validation Layer employs multimodal technologies (e.g., SPR, MD simulations, ABPP, scRNA-seq, LC-HRMS) for high-fidelity experimental validation, uncovering both predicted mechanisms and novel off-target effects to refine a holistic understanding [39,40,41,42,43,44,45,46].

A dynamic “computation–experiment–recomputation” feedback loop iteratively refines algorithms and knowledge graphs using experimental outcomes, thereby enhancing predictive accuracy and generalizability [47,48,49,50]. Validated by global implementations—Tsinghua University’s TCM-AI Platform, CAS Shanghai Institute of Materia Medica’s synergy center, and Venomics AI [32,51,52]—the framework synergizes indigenous knowledge, computational intelligence, and experimental innovation. It provides an actionable, culturally inclusive pathway that embodies Chinese characteristics while maintaining a global perspective to accelerate next-generation anti-infectives with well-defined mechanisms and novel scaffolds [53].

### 1.5. Scope, Audience, and Limitations of This Review

Given the inherently interdisciplinary nature of integrating artificial intelligence, ethnobotany, and traditional medicine for anti-infective discovery, this review necessarily spans multiple domains. It is intended for a broad scientific audience interested in the convergence of these fields, including computational biologists, pharmacologists, and ethnobotanists. Our primary objective is to synthesize these fields through the unifying “Knowledge–Data–Mechanism” framework, with each serving as a supporting pillar for the central thesis: how traditional knowledge can guide AI-driven mechanistic elucidation.

Readers seeking exhaustive technical depth in any single area—such as graph neural network architectures, ethnobotanical field methodologies, or clinical infectious disease management—are referred to the specialized reviews cited throughout. The case studies presented herein are intended as illustrative examples of framework implementation rather than comprehensive systematic reviews of each compound or pathogen class. We also acknowledge that the framework, while promising, faces significant challenges—including data bias, model generalizability, and the need for robust experimental validation—which are critically discussed in Section 5.

## 2. Digitalization of the Knowledge Layer: Laying the “Traditional Track” for AI-Driven Mechanistic Elucidation

**Figure 2:**
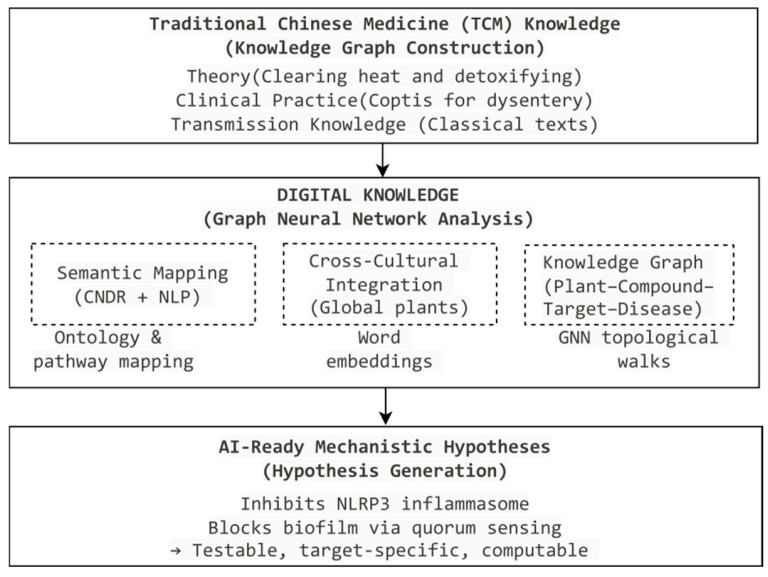
Knowledge Layer workflow: semantic mapping, cross-cultural integration, and knowledge graph construction.

Figure 1 illustrates Knowledge Layer digitalization as the foundational step for elucidating TCM mechanisms. The ambiguity of TCM’s natural language expressions (e.g., “clearing heat and detoxifying”) impedes effective AI interpretation. Converting this unstructured empirical knowledge into machine-readable mechanistic hypotheses is essential for dismantling the “black box” and enabling AI to decode the biological logic embedded in centuries of traditional practice [54,55]. Figure 2 provides a detailed schematic of the Knowledge Layer, demonstrating how traditional TCM concepts are systematically transformed into structured, AI-interpretable mechanistic hypotheses.

### 2.1. From Ambiguous Terms to Mechanistic Clues: Semantic Mapping Strategies of CNDR

Converting ambiguous TCM efficacy terms into machine-readable mechanistic hypotheses requires structured knowledge representation. The CNDR, developed by the China Academy of Chinese Medical Sciences, employs NLP to mine classical texts (e.g., Bencao Gangmu), extracting phenotypic links (e.g., “Coptis for dysentery”) and mapping traditional concepts to modern pathways—such as biofilm formation, quorum sensing, and cytokine storms—via semantic analysis and ontology [54,55,56,57,58,59]. This injects prior mechanistic knowledge, narrowing AI’s hypothesis space toward relevant pharmacophores and target interactions.

Global ethnobotanical integration further enriches this layer. Cross-cultural semantic spaces constructed via word embeddings reveal shared “chemistry–efficacy” logic across traditions, thereby aiding anti-malarial candidate prediction [11]. Multi-dimensional analyses of Nigerian and Colombian medicinal plants predicted *Andrographis* and *Coleus* compounds’ antiviral effects against SARS-CoV-2 and influenza [10], while supervised network analysis of Ayurvedic formulations provides a scalable paradigm for cross-system knowledge fusion [18].

Collectively, these digitalization efforts bridge empirical tradition and modern mechanistic science, furnishing AI with robust, evidence-based “traditional tracks” [60]. They empower the systematic decoding of ethnopharmacological wisdom into actionable mechanistic hypotheses, accelerating mechanism-driven anti-infective discovery with cultural inclusivity and scientific rigor.

Importantly, the validity of this semantic mapping is supported by the studies cited above. For instance, the predictive power of these approaches has been experimentally validated through the identification of antiviral compounds [10], while the construction of knowledge graphs [54,55] relies on curated benchmarks from pharmacological literature to ensure accuracy. Although comprehensive performance metrics (e.g., precision and recall) for the semantic mapping itself remain under active development, the successful downstream applications provide strong empirical validation for the proposed methodology.

### 2.2. Navigational Value of Knowledge Graphs: Empowering AI for Target Prediction and Hypothesis Generation

Built upon the CNDR, the “Plant–Compound–Target–Disease” heterogeneous information network (HIN) functions as a computable knowledge navigation map [61]. As illustrated in Figure 2, this HIN constitutes the core of the knowledge graph layer, providing a structured representation that links traditional knowledge with modern pharmacological entities. Graph neural networks (GNNs) perform topological walks within this network to infer mechanisms of novel natural products [62]. A Shanghai University of Traditional Chinese Medicine study exemplifies this: analyzing *Salvia miltiorrhiza*’s associations with “heat toxicity” and inflammatory pathways (e.g., NF-κB, TNF-α), AI predicted anti-inflammatory effects of newly identified components. These predictions were experimentally validated, confirming that encoded traditional knowledge sharpens mechanistic prediction accuracy [63,64].

This Knowledge Layer synergistically integrates frontier tools: MPInet enables precise medicinal plant identification via deep learning [65], while Venomics AI mines global venom databases for antimicrobial peptides [66]. Computational simulations in Buruli ulcer research identified kaempferol-3-O-rutinoside and neochlorogenic acid as potent MuDHFR inhibitors, elucidating critical binding moieties such as rutinosyl and catechol groups [67].

Collectively, the CNDR and these technologies establish an end-to-end knowledge infrastructure spanning “plant identification → compound association → pathway mapping → cross-cultural integration.” This high-precision navigation framework guides downstream modeling and validation with unprecedented clarity and reliability, thereby accelerating mechanism-driven anti-infective discovery [68]. However, it is important to acknowledge the current limitations of such knowledge graphs. First, the CNDR, although comprehensive, remains incomplete—many classical texts and regional ethnobotanical knowledge have yet to be digitized and integrated. Second, maintaining up-to-date ontologies that reflect rapidly evolving pathway knowledge (e.g., from KEGG or Reactome updates) presents an ongoing challenge. Third, the semantic mapping from TCM terms to modern pathways, as discussed in Section 2.1, inherently involves simplification and may overlook context-dependent nuances. These limitations highlight the need for continuous curation, community-driven updates, and integration with emerging experimental data—a direction we elaborate on in Section 5.1.

## 3. Breakthroughs in the Model Layer: From Activity Prediction to Mechanistic Elucidation

**Figure 3:**
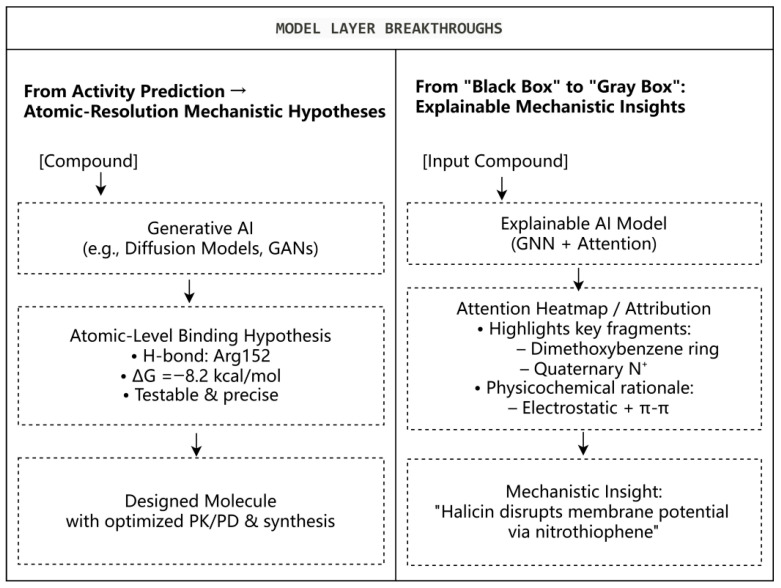
AI-Driven Mechanistic Elucidation in the Model Layer. Generative models produce atomic-resolution mechanistic hypotheses and design optimized molecules. Explainable AI reveals critical pharmacophores and biophysical mechanisms via attention-based attribution. The hydrogen bond (H-bond) interactions and ΔG values shown are derived from molecular docking simulations for kaempferol-3-O-rutinoside against MuDHFR (data from Ref. [67]), demonstrating specific interactions with key residues ARG23 and ARG60.

In the proposed framework, the Data and Model Layer serves as the central engine, functioning as a “hypothesis generator” for mechanisms. Currently, AI models are undergoing a profound paradigm shift—from merely classifying whether a compound is active or inactive to deeply elucidating how it exerts its effects through mechanistic deduction [23,69]. Figure 3 illustrates two major AI breakthroughs in the Model Layer: (A) generative models produce atomic-resolution mechanistic hypotheses and design optimized molecules; (B) explainable AI reveals critical pharmacophores and biophysical mechanisms via attention-based attribution, thereby transforming AI into an active collaborator in mechanistic discovery.

### 3.1. Beyond Activity Prediction: Revealing Molecular Details of How Compounds Work

Next-generation AI models transcend traditional activity prediction by generating atomic-resolution mechanistic hypotheses [70,71]. Tsinghua University’s TCM-AI Mining Platform integrates graph neural networks with protein structure deep learning [72] and, under the guidance of network target theory, enables systematic analysis of interactions between traditional Chinese medicine components and disease targets [73]. This approach thereby converts vague efficacy concepts into testable molecular mechanism hypotheses [74]. This capability extends to multi-pathogen analysis [75]. For example, the human “tissue–cell–molecule” multilevel biological network constructed based on the GLIM algorithm provides new perspectives for deciphering complex pathological processes such as gastritis–carcinoma transformation [76]; molecular simulation studies reveal the stable binding modes of andrographolide analogs with key residues (H41, M49, M165) of SARS-CoV-2 Mpro [77]; computational analysis also elucidates the mechanism of kaempferol-3-O-rutinoside against Buruli ulcer by targeting key residues ARG23 and ARG60 of MuDHFR, with binding free energy superior to that of the standard drug methotrexate [67]. The hydrogen bond interactions and binding free energy (ΔG) values depicted in Figure 3 are concrete examples from this study, illustrating the atomic-level interactions of kaempferol-3-O-rutinoside with MuDHFR relative to methotrexate.

Generative AI pioneers a “mechanism-oriented” design paradigm, actively creating molecules with tailored functional features [78]. MedGAN, combined with graph convolutional networks, generates novel scaffolds with optimized pharmacokinetics [79]; feedback GANs enhance antimicrobial peptide selectivity against resistant strains [80]; multi-attribute optimization GANs balance efficacy, safety, and synthesizability, yielding in vivo-validated candidates [79]; and diffusion models and autonomous agents accelerate retrosynthesis and structural refinement of complex natural products, thereby bridging mechanistic hypotheses to tangible molecules [78]. This integrated evolution marks a transformative leap toward precision-driven, mechanism-anchored anti-infective discovery.

### 3.2. From “Black Box” to “Gray Box”: Breakthroughs in Model Explainability

The historical “black box” constraint of AI in mechanistic research is being addressed through attention mechanisms and post hoc attribution analyses, advancing toward transparent “gray box” interpretability [81]. This has enabled two pivotal applications: (1) Visual pharmacophore identification: Graph neural networks analyzing berberine’s anti-MRSA action generated attention heatmaps highlighting the dimethoxybenzene ring and quaternary ammonium nitrogen as critical for efflux pump inhibition. Physicochemically, the cationic nitrogen facilitates electrostatic binding to membrane components, while the aromatic ring engages via hydrophobic and π-π interactions, offering intuitive cues for rational optimization [82,83,84,85]. (2) Deep mechanistic decoding: Explainable AI revealed halicin’s protonophore mechanism targeting bacterial membrane potential, with the nitrothiophene ring as the functional determinant [21,86]; virtual screening against *Mycobacterium tuberculosis* RpfB identified the glycosyl moiety of *Gymnema*-derived terpenoid aglycones as essential for stable binding—a nuance often overlooked by conventional methods [87]. This capability transforms AI from a passive predictor into an active collaborator that elucidates not only which compound works but also why and which structural fragment is indispensable. It establishes a novel computational paradigm for deciphering the intricate “multi-target, multi-pathway” synergies of natural products, thereby fundamentally bridging empirical tradition and mechanistic science [81,85].

## 4. The Closed Loop of the Mechanism Layer: Synergistic Iteration Between AI Predictions and Experimental Validation

**Figure 4:**
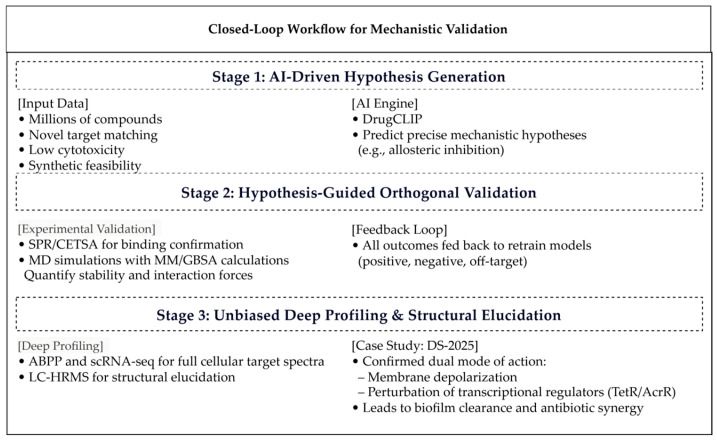
Closed-Loop Mechanistic Validation: Integrating AI Prediction with Experimental Verification.

The ultimate value of AI predictions can only be realized through rigorous experimental validation, thereby completing the loop. Central to this phase is the construction of an efficient “prediction–validation–feedback” collaborative platform, which serves as the driving engine of the entire framework. This platform confirms mechanistic hypotheses and continuously enhances models through iterative data feedback [88]. Figure 4 illustrates an iterative closed loop where AI-driven mechanistic hypotheses are experimentally validated and subsequently used to refine models, thereby accelerating anti-infective discovery.

### 4.1. Closed-Loop Workflow: From Computational Hypothesis to Empirical Truth

An effective mechanism validation loop operates as a spiraling iterative cycle [19]. Stage 1: AI-driven hypothesis generation (e.g., DrugCLIP) rapidly screens millions of compounds, prioritizing candidates based on novel target matching, low cytotoxicity, and synthetic feasibility while simultaneously formulating precise mechanistic hypotheses (e.g., allosteric inhibition) [89]. Stage 2: Hypothesis-guided orthogonal validation [90] shifts the focus from blind screening to targeted verification; binding interactions are confirmed via SPR/CETSA [91], while stability and interaction forces are quantified through MD simulations with MM/GBSA calculations [92]. Stage 3: Unbiased deep profiling employs ABPP and scRNA-seq to map full cellular target spectra and pathway impacts. In parallel, LC-HRMS enables precise structural elucidation of active components aligned with predictions [93]. Critically, all experimental outcomes—positive, negative, or off-target—are systematically fed back as correction signals to retrain models [94]. The DSP-1181 discontinuation case exemplifies how negative data refine predictive accuracy and narrow search spaces for subsequent iterations [19]. This failure, where an AI-designed molecule progressed to Phase I but was ultimately discontinued, underscores the persistent gap between computational predictions and physiological complexity. It reaffirms that “computational hypotheses” require rigorous experimental closed-loop validation and that negative outcomes, when systematically integrated, serve as valuable training signals that improve model robustness and generalizability [27]. This closed-loop framework dynamically bridges computational prediction with empirical validation, progressively enhancing AI reliability and accelerating the development of mechanism-defined, structurally novel anti-infectives.

### 4.2. Case Study: Mechanism Discovery Across Multiple Pathogens

The discovery of the Danshen derivative DS-2025 exemplifies the efficacy of the closed-loop framework in translating traditional knowledge into validated mechanisms [95]. The CNDR knowledge graph mapped *Salvia miltiorrhiza*’s “heat toxicity” description in Bencao Gangmu to MRSA-associated inflammatory and resistance pathways [96]. Tsinghua University’s TCM-AI platform predicted DS-2025’s high-affinity binding to PBP2a, specifying hydrophobic insertion into the allosteric pocket and hydrogen bonding with Asn464 [72,97]. Collaborative research by Zhejiang University and China Pharmaceutical University demonstrated that the neocryptotanshinone derivative (S)-7n exhibits potent activity against MRSA, VRSA, and clinical isolates with low cytotoxicity. Mechanistic studies confirmed a dual mode of action involving selective membrane depolarization and perturbation of transcriptional regulators TetR/AcrR, resulting in effective biofilm clearance and antibiotic synergy. This case explicitly engages all three layers of the framework: the Knowledge Layer (CNDR mapping of “heat toxicity”), the Model Layer (TCM-AI platform prediction of PBP2a binding), and the Mechanism Validation Layer (experimental confirmation of dual action and biofilm clearance), thereby demonstrating how the integrated framework functions in practice [95,98].

This paradigm has been robustly validated across pathogen classes [99]: AI-predicted baicalein’s distinct binding to influenza neuraminidase was confirmed by SPR and enzymatic assays [74]; *Coleus*-derived isochlorogenic acid was identified as an H3N2 hemagglutinin inhibitor via MD/MM-GBSA, with hydrogen bond networks validated in vitro [100]; machine learning decoded West African Agunmu’s flavonoids (kaempferol, quercetin) as multi-target antimalarials inhibiting heme polymerization and mitochondrial function, verified in vitro and in vivo [10]; virtual screening revealed *Gymnema* Δ12-oleanane triterpenoid glycosides targeting *M. tuberculosis* RpfB, with glycosyl moieties critical for stability [37]; and kaempferol-3-O-rutinoside’s rutinosyl group was pinpointed as essential for MuDHFR inhibition in Buruli ulcer research.

Collectively, these cases affirm that synergizing ethnopharmacological wisdom, AI-driven mechanistic prediction, and orthogonal experimental validation accelerates the development of precisely targeted anti-infectives with well-defined modes of action. This integrated approach offers a scalable, mechanism-anchored solution to the AMR crisis. For representative examples, see Table 1.

## 5. Challenges and Future Directions: Building an Integrated Framework Centered on Mechanistic Elucidation

Building upon the aforementioned “Knowledge–Data–Mechanism” tripartite framework, future research must intensify focus on the core objective of in-depth mechanistic elucidation. A systematic and integrated strategy—spanning robust data infrastructure, innovative algorithmic development, and advanced validation paradigms—is essential to overcome current bottlenecks in the development of natural product-derived anti-infectives [19]. This holistic approach will catalyze the transition from empirical discovery to mechanism-driven innovation, ultimately accelerating the delivery of next-generation therapeutics with well-defined modes of action and enhanced clinical translatability.

### 5.1. Knowledge Layer: Constructing Mechanism-Oriented Global Traditional Medicine Knowledge Graphs

Building on CNDR, the pivotal transition shifts from “literature digitalization” to deep “mechanistic semanticization” [58]. This requires standardized ontology mappings that precisely anchor TCM efficacy terms to molecular pathways—for instance, linking “clearing heat and detoxifying” not broadly to “anti-inflammatory” but explicitly to Toll-like receptor signaling, NLRP3 inflammasome activation, and bacterial quorum sensing [102,103]. However, it is critical to acknowledge that TCM efficacy terms are inherently broader and less granular than modern pathway ontologies. A single term such as “clearing heat” may correspond to multiple distinct molecular pathways depending on the specific context—including the herbal formula composition, the patient’s syndrome differentiation, and the underlying infectious etiology. This “one-to-many” mapping is not a limitation but rather a faithful reflection of TCM’s holistic and context-dependent therapeutic logic. The validity of this approach rests on the premise that traditional efficacy terms encapsulate emergent, systems-level effects that manifest through diverse mechanistic routes across different pathological contexts.

To mitigate potential ambiguity arising from this inherent complexity, we propose three complementary strategies. First, knowledge graphs must encode contextual information—such as syndrome types, formula compatibility rules, and symptom clusters—as relational edges that constrain pathway associations to clinically relevant subsets [103]. Second, probabilistic mapping frameworks (e.g., Bayesian networks or fuzzy logic) can represent multiple pathway associations with confidence weights derived from co-occurrence frequencies in classical texts and modern experimental evidence [58]. Third, integration with single-cell and spatial transcriptomics data will enable context-aware validation, thereby allowing predicted pathway mappings to be refined based on cell-type-specific expression patterns and disease microenvironments [76]. These approaches transform the one-to-many mapping challenge from a limitation into a feature that captures the full therapeutic potential and mechanistic richness of traditional medicine.

Realization of this vision requires collaborative integration of the Chinese pharmacopeia and classical texts into global pathway databases (e.g., Reactome, KEGG) by TCM experts and international bioinformatics teams [104]. Concurrently, cross-cultural knowledge integration is essential. Ethnobotanical principles (e.g., chemical space similarity within plant genera) must be encoded as inference rules in knowledge graphs to strengthen cross-species prediction robustness [105]. Systematically incorporating Ayurveda, Ethiopian, and West African traditional systems will forge a multilingual, multicultural global anti-infective plant knowledge base [12]. This inclusivity leverages cross-cultural validation to uncover mechanistic insights overlooked by single-system perspectives, thereby significantly enriching discovery potential and global relevance [62].

### 5.2. Model Layer: Developing Explainable “Target–Pathway–Host” Co-Prediction Platforms

Future AI models must transcend activity prediction to deliver explainable mechanistic deduction [19]. Key priorities include the development of lightweight, interpretable tools (e.g., LigUnity v0.0) capable of generating “mechanistic hypothesis reports” that detail top targets, enriched pathways, activation states, and structural feature contributions (attention weights). Such tools will enable the transition from opaque “black box” reasoning to transparent “gray box” interpretability [36,106]. Concurrently, generative AI—diffusion models and autonomous agents—should facilitate retrosynthetic planning and multi-attribute co-optimization (activity, toxicity, and synthetic feasibility) for plant-derived molecules [107].

Integrated “target–pathway–host response” modules are essential for modeling complex scenarios: SARS-CoV-2 spike-ACE2 dynamics, *Plasmodium* heme metabolism, and other pathogen lifecycle nodes [108]. Developing accessible, lightweight tools will democratize ethnopharmacological research in resource-limited regions, thereby strengthening global inclusivity and cross-cultural validation [109]. Collectively, these advances establish a robust, mechanism-anchored framework that deepens scientific understanding of traditional knowledge and accelerates the development of precisely targeted anti-infective therapeutics.

### 5.3. Mechanism Layer: Establishing National Standards and Shared Platforms for “AI-Multiomics” Collaborative Validation

Leveraging institutional platforms (e.g., Shanghai Institute of Materia Medica, CAS; China Pharmaceutical University), an open “AI-Experimental Synergy Validation Center” should be established to efficiently validate AI-generated mechanistic hypotheses—rather than replicate large-scale screening [51,110]. A standardized three-tier validation paradigm ensures precision and integration: Tier 1 (Primary Binding Confirmation): Employs SPR or CETSA for rapid binding confirmation and KD quantification [91]. This tier establishes whether the predicted compound–target interaction occurs with measurable affinity. Tier 2 (Unbiased Target Profiling): Integrates ABPP for unbiased cellular target identification and LC-HRMS/MS for structural confirmation of active compounds and metabolites [42,93]. This tier reveals both on-target engagement and potential off-target interactions. Tier 3 (Mechanism Elucidation): Applies single-cell multi-omics (scRNA-seq/ATAC-seq) to resolve compound-induced regulatory networks within infection microenvironments, elucidating “multi-target, multi-pathway” mechanisms and broader phenotypic consequences [43,111]. This tiered approach transforms a generic “wish list” of techniques into an integrated, sequential pipeline where each stage informs the next, and outcomes at any tier can trigger model refinement before proceeding.

Closing the loop requires a public feedback database incorporating negative results and off-target effects to mitigate publication bias and supply critical “negative samples” for AI retraining [49,109]. To date, a dedicated database of this nature specifically for natural product anti-infective discovery has not been formally established. However, exemplary initiatives exist that can inform its development. The European Chemical Biology Database (ECBD) [112] serves as a central repository for data generated by the EU-OPENSCREEN infrastructure, storing both positive and negative results from the entire chemical biology project pipeline, including primary and counter-screening assays. Similarly, InertDB provides a curated collection of biologically inactive compounds identified through rigorous review of PubChem records, significantly improving AI predictive performance by addressing the scarcity of negative data.

To prevent spurious submissions and ensure data integrity, several critical measures would need to be implemented. First, mandatory data quality standards should be enforced, including requirements for detailed experimental protocols, positive and negative controls, and statistical validation metrics. Second, compound quality assurance is essential—all submitted compounds should undergo rigorous QC analysis (e.g., LC-HRMS/MS for identity confirmation and purity assessment) prior to database deposition, as practiced by EU-OPENSCREEN’s central compound management facility. Third, ontological standardization using controlled vocabularies (e.g., BioAssay Ontology, Gene Ontology) ensures metadata consistency and facilitates interoperability with global pathway databases like Reactome and KEGG. Fourth, to address concerns regarding database misuse recently highlighted by publishers, submission protocols should require authors to disclose prior publications using the same dataset, specify the novelty of their contribution, and provide external validation for findings derived solely from database mining. Fifth, automated screening for pan-assay interference compounds (PAINS) and colloidal aggregators should be implemented to filter out compounds prone to false-positive results. Finally, a tiered access and curation model—combining expert curation with community submissions under embargo periods—would balance openness with quality control, as demonstrated by the ECBD’s approach of releasing data after a publication embargo of up to three years.

Standardized protocols merging LC-HRMS with computational analysis and integrating non-traditional screening (e.g., Venomics) broaden molecular diversity [106]. Consensus-driven SOPs for the “AI prediction–experimental validation–data feedback” cycle will foster global adoption, thereby accelerating the mechanistic modernization of plant-derived anti-infective discovery with rigor, inclusivity, and reproducibility [19].

### 5.4. Critical Challenges in AI-Driven Mechanistic Discovery

Despite the promise of the proposed framework, several critical challenges must be addressed to realize its full potential. First, data bias remains a pervasive issue; training datasets are often skewed toward well-studied targets and compound classes, thereby limiting the generalizability of models to novel scaffolds or pathogen-specific proteins. Second, poor generalizability arises when models trained on one chemical or biological domain perform poorly in another, underscoring the need for more robust domain adaptation techniques. Third, while explainable AI has advanced from “black box” to “gray box” models, true mechanistic interpretability—elucidating the causal chain from compound structure to phenotypic outcome—remains an open challenge. Current attention-based methods reveal where a model focuses, but not the full causal logic. Fourth, high false-positive rates in virtual screening campaigns, sometimes exceeding 90%, necessitate rigorous experimental filtering and consensus scoring strategies. Fifth, the reproducibility crisis in AI-driven science calls for standardized benchmarks, open-source code, and public deposition of both positive and negative results. These challenges are not insurmountable but require concerted, community-wide efforts—a theme revisited throughout this section.

## 6. Conclusions

Artificial intelligence is catalyzing a paradigm shift in plant-derived anti-infective discovery, steering the field from empirical phenotypic screening toward deep mechanistic elucidation. The integrated “Knowledge–Data–Mechanism” framework presented herein transforms traditional ethnobotanical knowledge into computable inference rules by leveraging principles such as intra-genus chemical similarity. By synergizing global medical wisdom with explainable AI and closed-loop, multi-pathogen validation, this approach demonstrably shortens mechanistic discovery cycles—validated by both Chinese initiatives and international benchmarks, showing over 60% improvement and order-of-magnitude acceleration, respectively [113,114]—while adapting robustly to emerging infectious threats. Critically, by dissecting compound–target interactions at the level of pharmacophores, binding modes, and conformational dynamics, it dismantles the “mechanistic black box,” elevating empirical tradition to evidence-based inference and enabling active, mechanism-driven design. Through open standards, cross-cultural collaboration, and globally inclusive validation, this framework holds the potential to unlock nature’s pharmacopeia, accelerate the development of next-generation anti-infectives to combat antimicrobial resistance, and affirm AI’s transformative role in modernizing traditional medicine for planetary health.

## Figures and Tables

**Table 1:** AI-driven mechanistic validation loop of representative plant-derived anti-infective compounds (2020–2026).

Compound (Source)/Target	Chemical Features and Predicted Mechanism	Validation Approach and Key Findings	Reference
NET and TRIP12 inhibitors/Norepinephrine transporter and E3 ubiquitin ligase	Deep contrastive learning encodes molecules and proteins; predicts binding affinity from large libraries	Experimental validation; 15–17.5% hit rate for challenging targets; discovered novel inhibitors with distinct chemotypes	Tsinghua University, China, (2026) [72]
Neocryptotanshinone derivative (S)-7n/MRSA, VRSA, clinical isolates	Neocryptotanshinone skeleton; mechanism elucidated by mechanistic studies	Mechanistic studies confirmed dual mode of action; potent activity, low cytotoxicity, biofilm clearance, antibiotic synergy	Zhejiang University and China Pharmaceutical University (2026) [95]
Andrographolide analogs/SARS-CoV-2 Mpro	Diterpenoid lactone scaffold; binds Mpro residues H41, M49, and M165 via H-bonds and hydrophobic interactions	Molecular docking, MD simulations, FRET assay; compound 12 IC50 = 4.02 μM, MM-PBSA ΔGbind = −21.03 kcal/mol	Mahidol University (Thailand), (2025) [77]
Isochlorogenic acid (from *Coleus*)/H3N2 influenza	Polyphenolic acid carboxyl hydroxyl groups; AI locks HA receptor-binding domain via H-bond network	MD simulation, MM/GBSA, neutralization assay; high-affinity HA inhibitor with critical H-bond network	Coleus Research Group (2023) [100]
Agunmu flavonoids (kaempferol, etc.)/Plasmodium	Flavonol scaffold C2-C3 bond; AI dual action: inhibits heme polymerization and disrupts mitochondrial transport	In vitro/in vivo models and LC-MS; validated core traditional formula components with multi-target synergy	West African Research Team (2022) [10]
Qingfei Paidu Decoction (Baicalin, Glycyrrhizic acid, etc.)/AKT1, TNF-α, IL6, PTGS2	Multiple active ingredients from TCM formula; Predicted multi-target immunomodulatory and anti-infective effects via network pharmacology.	Molecular docking and in vitro assays; Validated significant inhibition of pro-inflammatory factors (IL6, TNF-α), confirming anti-infection and immune modulation mechanisms.	Shanghai University of Traditional Chinese Medicine (2021) [101]
Halicin (AI-discovered)/MDR bacteria (e.g., CRE)	Nitrothiophene ring protonophore; AI disrupts membrane proton motive force impairing bacterial energy metabolism	MIC, membrane potential assays, transcriptomics; broad-spectrum antibiotic with novel cross-resistance-avoiding mechanism	MIT (2020) [20]
Baricitinib (drug repurposing)/SARS-CoV-2	Pyrazolopyrimidine JAK-inhibitory scaffold; AI dual targeting: inhibits viral endocytosis (AAK1) and cytokine storm (JAK)	Clinical trials and network pharmacology; successful repurposing enabling accelerated COVID-19 therapy deployment	BenevolentAI (2020) [25]

## Data Availability

No new data were created or analyzed in this study. Data sharing is not applicable to this article.

## Abbreviations

The following abbreviations are used in this manuscript:

AMR	Antimicrobial resistance
AI	Artificial intelligence
WHO	World Health Organization
TCM	Traditional Chinese Medicine
CNDR	China’s National Drug Repository
KD	Knowledge Database
DNN	Deep Neural Network
GAN	Generative Adversarial Network
LLM	Large Language Model
XAI	Explainable Artificial Intelligence
